# Left Atrial Volume and Phasic Function Assessed by 4D Auto LAQ Echocardiography in Treatment-Naive, Newly Diagnosed Type 2 Diabetes Mellitus Patients Without Hypertension or Obesity

**DOI:** 10.3390/jcdd13030131

**Published:** 2026-03-10

**Authors:** Lin Li, Miao Li, Hong Ran, Ling-Ling Fang, Qian-Shan Ding, Ping-Yang Zhang

**Affiliations:** Department of Cardiovascular Ultrasound, Nanjing First Hospital, Nanjing Medical University, Nanjing 210000, China; lilin1990mu@163.com (L.L.); lim0806@foxmail.com (M.L.); ranhong1209@hotmail.com (H.R.); fanglingling753@163.com (L.-L.F.); dingqs@outlook.com (Q.-S.D.)

**Keywords:** four-dimensional echocardiography, left atrial function, strain, diabetes mellitus

## Abstract

(1) Background: Our aim was to evaluate left atrial (LA) volumes and function in patients with newly diagnosed, treatment-naive type 2 diabetes mellitus (T2DM) using Four-Dimensional Automated Left Atrial Quantificative (four-dimensional auto LAQ) analysis and to explore the independent factors influencing left atrial function in diabetic patients. (2) Method: A total of 62 treatment-naive, newly diagnosed T2DM patients without hypertension or obesity and 50 healthy controls were prospectively enrolled in the study. All participants underwent laboratory analyses, routine echocardiography and 4D LAQ assessment. The parameters were compared between the two groups, and independent factors influencing left atrial function in diabetic patients were investigated through univariate and multivariate linear regression analyses. (3) Results: Despite no significant difference in LA end-systolic anteroposterior diameter between groups, LA volume parameters (LAVmax, LAVmin, LAVpreA, and LAVmaxI) were significantly increased in T2DM patients (all *p* < 0.05). Regarding LA strain, reservoir and conduit function were significantly impaired in T2DM patients, as reflected by lower LASr, LAScd, LASr-c, and LAScd-c (all *p* < 0.05). Conversely, circumferential contractile strain (LASct-c) was significantly higher in the T2DM group (*p* = 0.029), while longitudinal contractile strain (LASct) did not differ significantly between groups (*p* = 0.146). Multivariate analysis revealed that HbA1c and E/e’ ratio were independently associated with multiple LA strain parameters (all *p* < 0.05). (4) Conclusion: Newly diagnosed, treatment-naive patients with T2DM exhibited increased LA volumes, decreased left atrial ejection fraction (LAEF), and impaired reservoir and conduit functions, accompanied by a compensatory increase in contractile function. Furthermore, HbA1c and E/e’ demonstrated an independent correlation with 4D strain parameters. 4D Auto LAQ echocardiography may serve as a sensitive tool for early detection of diabetic atrial myopathy.

## 1. Introduction

Type 2 diabetes mellitus (T2DM) is one of the most prevalent chronic diseases in the general population [1]. The metabolic and microvascular changes in T2DM contribute to cardiac dysfunction and increase the risk of heart failure with preserved ejection fraction and atrial fibrillation [2]. Assessing myocardial deformation offers valuable insights into subtle changes in cardiac function that may predict cardiovascular morbidity and mortality [3].

Early changes in left ventricular (LV) function in T2DM patients have been extensively investigated [4,5,6]. Meanwhile, the assessment of left atrial function is of growing interest, as recent studies have demonstrated that LA phasic function is a strong and independent predictor of cardiovascular events in a large population of patients, both with and without heart failure [7,8]. However, current knowledge regarding changes in atrial phasic function and mechanics in T2DM patients remains insufficient.

Left atrial strain analysis, particularly through speckle-tracking echocardiography (STE), has been proposed as an alternative method for assessing LA dysfunction [9]. However, despite its potential to provide valuable diagnostic and prognostic insights [10], the clinical applications of STE are limited because the available software packages are predominantly designed for left ventricular analysis [11]. The thin-walled structure of LA, the irregular arrangement of LA myocytes, the presence of LA appendage and pulmonary veins, as well as variability between different vendors, complicate the reliable tracking and precise subdivision of LA segments [12].

The recently developed Four-Dimensional Automated Left Atrial Quantification (4D Auto LAQ) Volume-Strain tool is a specialized analysis instrument designed exclusively for the left atrium. It utilizes three-dimensional volume data to assess both the volume and LA ejection fraction (LAEF), as well as the longitudinal and circumferential strains of the left atrium [13]. This is particularly relevant given the intricate intermingling of longitudinal and circumferential bundles within the left atrial wall [14]. Initial studies have demonstrated its feasibility and reproducibility, although results may vary between different software vendors [15,16]. However, whether the combination of longitudinal and circumferential strain improves the detection of LA dysfunction in T2DM patients is unknown.

Moreover, previous studies on LA function in T2DM have mostly included patients with established disease, many of whom were on glucose-lowering medications. These medications, particularly SGLT2 inhibitors and GLP-1 receptor agonists, have known cardioprotective effects that may confound the assessment of diabetes-related atrial remodeling. Whether LA dysfunction occurs in the earliest stage of diabetes, before any pharmacological intervention, remains unclear.

Therefore, this study focused on newly diagnosed, treatment-naive T2DM patients without hypertension or obesity. The primary purpose of this study was to use 4D Auto LAQ echocardiography to evaluate LA volumes and phasic function in this unique population and to identify factors independently associated with LA dysfunction.

## 2. Materials and Methods

### 2.1. Study Population

The present investigation included 80 normotensive subjects (blood pressure < 140/90 mmHg) with type 2 diabetes mellitus and 50 controls matched in age and gender. Diabetes was diagnosed according to the current guidelines, defined as a fasting blood glucose level ≥ 7.0 mmol/L, or a 2 h plasma glucose level ≥ 11.1 mmol/L during an oral glucose tolerance test (OGTT), or HbA1c level ≥ 6.5%. Importantly, only patients with newly diagnosed T2DM who had not yet received any glucose-lowering medications (including metformin, SGLT2 inhibitors, GLP-1 receptor agonists, or insulin) were included. For the definition of normotension, we adopted the threshold of <140/90 mmHg, which aligns with the diagnostic criteria for hypertension established in the 2023 Chinese Guidelines for the Prevention and Treatment of Hypertension.

**Figure 1:**
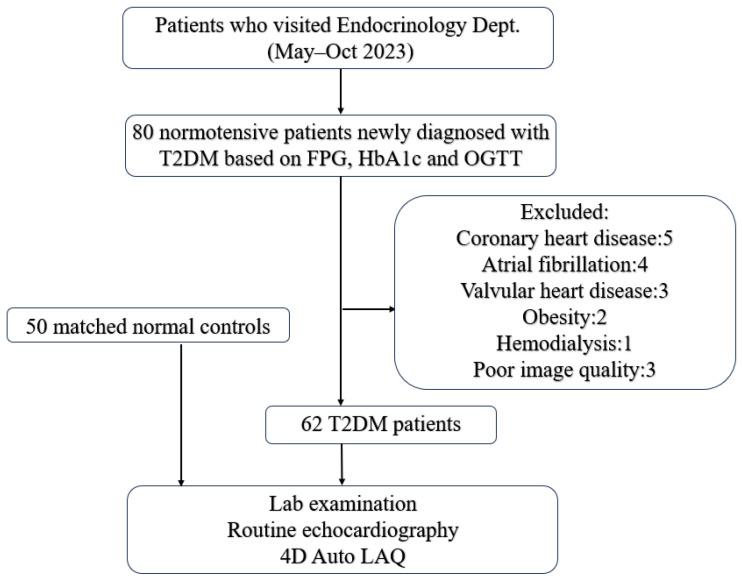
Flow chart.

Subjects exhibiting symptoms or signs of cardiovascular disease (such as arterial hypertension, heart failure, myocardial infarction, significant valvular disease, atrial fibrillation, congenital heart disease), obesity (BMI ≥ 30 kg/m^2^), asthma, chronic obstructive pulmonary disease, neoplastic disease, liver cirrhosis, kidney failure, or those with poor echocardiographic image quality were excluded from the study. Consequently, 62 patients were included in the final study (Figure 1).

Anthropometric measures (height and weight) and laboratory analyses (fasting glucose level, HbA1c, total cholesterol, triglycerides, and C reactive protein) were conducted for all subjects included in the study. Body mass index (BMI) and body surface area (BSA) were calculated for each patient.

The study was approved by the local Ethics Committee of Nanjing First Hospital, Approval No. 2023048, and informed consent was obtained from all the participants.

### 2.2. Echocardiography Image Acquisition and Analysis

All subjects underwent transthoracic echocardiographic examinations conducted by an experienced cardiac sonographer at rest, utilizing a commercially available ultrasound system (VividTM E95 Version 203, GE Medical Systems, Horten, Norway) equipped with an M5Sc transducer for two-dimensional echocardiography (2DE) and a 4Vc transducer for four-dimensional echocardiography (4DE). During the examinations, subjects were monitored with a stable electrocardiogram (ECG) of good quality, with a clearly visible P wave.

### 2.3. Two-Dimensional and Doppler Assessment

Traditional 2D evaluation was performed using maintained images. Left ventricular ejection fraction (LVEF) was measured by the modified biplane Simpson method. Left ventricular diastole diameter and left atrial systole diameter were observed and measured in the long-axis view. Peak early diastolic (E) and late diastolic (A) filling velocities were assessed through the transmittal flow measured by pulsed-Doppler ultrasound. Peak mitral diastolic annular velocities (e′ and a′) were obtained in the apical 4-chamber view by pulsed tissue Doppler echocardiography, averaging the septal and lateral velocities. Left ventricle filling pressure was evaluated according to E/e′ ratio.

### 2.4. Four-Dimensional Volume and Strain Assessment

Following conversion to the 4D volume transducer with an apical 4-chamber view, the LV and LA walls were clearly displayed. Images of three cardiac cycles were acquired with an angle (70 to 80 degrees) × (70 to 80 degrees), with a 4D frame rate exceeding 40% of the heart rate. Upon entering the 4D mode, patients were instructed to hold their breath at the end of either inhalation or exhalation. A minimum of four datasets were obtained for each participant, with the three best-quality datasets selected for offline analysis. Datasets that exhibited missing portions of the LA, blurred endocardial boundaries, or significant stitch artifacts were excluded. During echocardiographic acquisition, all patients were in sinus rhythm. High-quality images were collected to enhance the accuracy and repeatability of the measurements.

**Figure 2:**
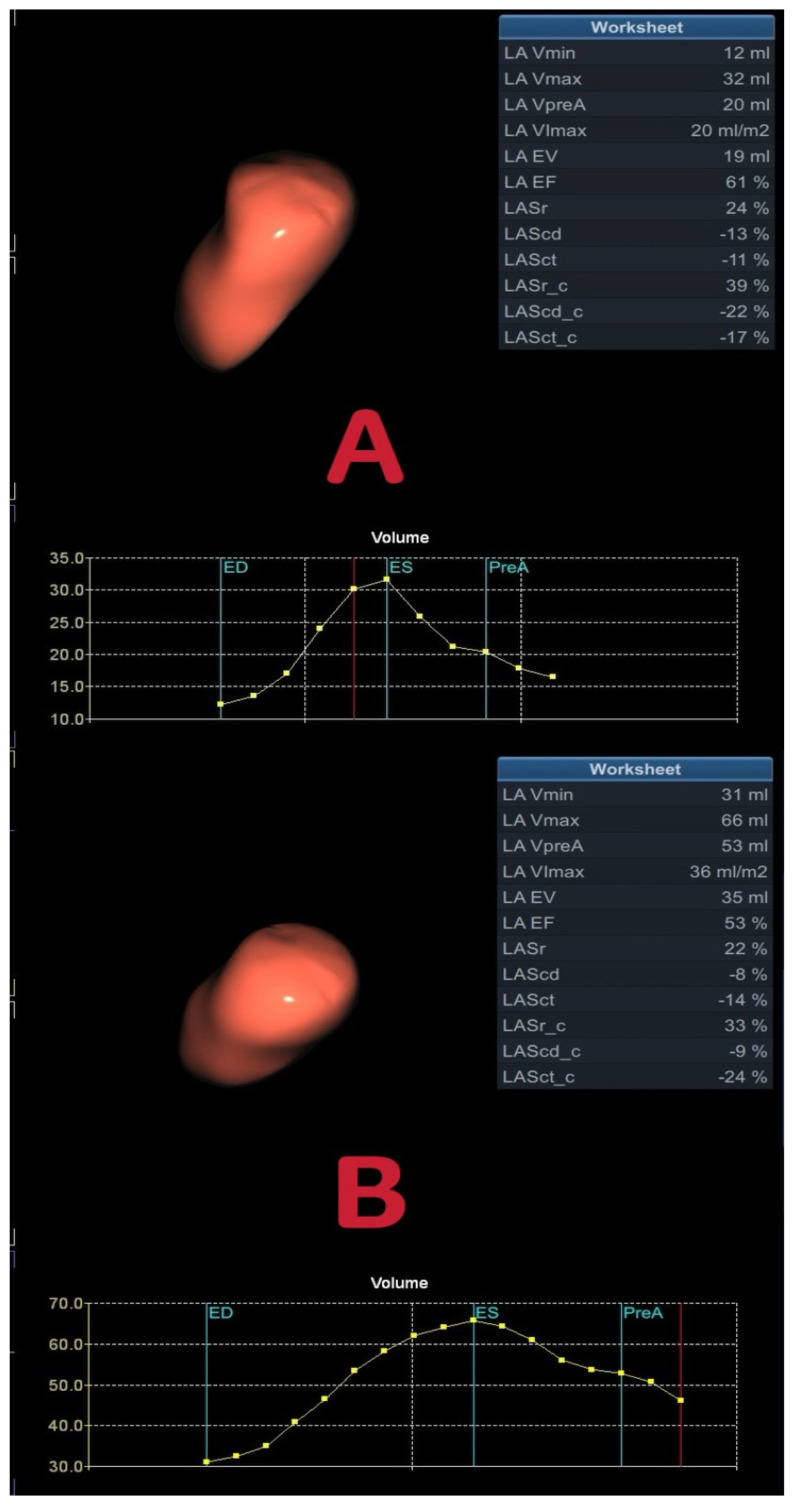
An example of 4D LAQ. (**A**): Control, (**B**): T2DM patient.

Images were imported into the EchoPAC 203 software (GE Healthcare) for analysis. The volume 4D auto LAQ measurement mode was activated, allowing for the setting of landmarks and the adjustments of the mitral valve center on each plane at end-systole, thereby clearly displaying the mitral annulus, wall and roof. The endocardial mesh was employed to measure both end-systolic and end-diastolic volumes, as well as to assign strain on every plane. Images were acquired with the reference point set to the onset of the QRS-complex, facilitating the acquisition of reservoir, conduit, and contractile function. The results stage provided an overview of various parameters regarding LA volumes and function as follows: LA minimum volume (LAVmin); LA maximum volume (LAVmax); LA volume at the onset of atrial contraction (LAVpreA); LA ejection volume (LAEV); LA ejection fraction (LAEF); LA longitudinal strain during reservoir (LASr), conduit (LAScd) and contraction (LASct) phases, as well as LA circumferential strain during reservoir (LASr_c), conduit (LAScd_c) and contraction (LASct_c) phases. The LAVmax index (LAVmI) was calculated by normalizing LAVmax to body surface area (BSA) (shown in Figure 2).

### 2.5. Sample Size Estimation

Based on a two-independent-sample Student’s *t* test, assuming a mean difference in LASr of 4% with a standard deviation of 7% (derived from the pre-experiment of this study), an alpha level of 0.05 and a power of 0.80, the minimum required sample size was 48 per group. Our final sample (62 T2DM, 50 controls) exceeds this threshold and is therefore adequately powered for the primary comparison.

### 2.6. Repeatability

To assess the repeatability of measurements, 10 patients were randomly selected, and the LA strain parameters were measured by a single observer on two separate occasions to evaluate intraobserver variability. Additionally, another observer independently assessed the same parameters for the same patients to determine interobserver variability. Both observers were blinded to each other’s results. The repeatability was analyzed using the intraclass correlation coefficient (ICC) and variable of coefficient (COV).

### 2.7. Statistical Analysis

All statistical analyses were conducted using SPSS version 19.0 (SPSS, Inc., Chicago, IL, USA) and Microsoft Excel (Microsoft Corporation, Redmond, WA, USA). Data were presented as means ± standard deviations (SD) for continuous variables and as numbers or percentages for categorical variables. Between-group comparisons for continuous data were performed using an independent-sample Student’s *t*-test, while categorical data comparisons were conducted using the chi-square test. The independent factors influencing left atrial function in diabetic patients were explored through univariate and multivariate linear regression analysis. A *p*-value of less than 0.05 was deemed statistically significant.

## 3. Results

### 3.1. Comparison of Clinical Characteristics

There were no significant differences in age and gender distribution between the T2DM and control groups. Patients with T2DM exhibited a higher BMI compared to the controls. As expected, the levels of fasting glucose and HbA1c were elevated in the T2DM patients. Additionally, the triglycerides, cholesterol and LDL levels were higher, while HDL levels were lower in the T2DM group. The serum creatinine levels were comparable between the observed groups (Table 1).

### 3.2. Comparison of Conventional Echocardiography Parameters

We observed no significant differences in left ventricular ejection fraction (LVEF), left ventricular end-diastolic diameter (LVEDD), stroke volume (SV), left atrial diameter (LAD), interventricular septal thickness (IVST), and left ventricular posterior wall thickness (LVPWT). However, we found that the average E/A value and the average e’/a’ values of T2DM patients were significantly lower than those in the control group. Additionally, the average E/e’ values in T2DM patients were significantly higher than those in the control group (Table 2).

### 3.3. Comparison of 4D Auto LAQ Echocardiography Parameters

The left atrial volume parameters, including LAVmax, LAVmin, LAVpreA, and LAVmaxI, were significantly elevated in the T2DM groups compared to the control group. In contrast, LAEF was significantly lower in the T2DM groups than in the control group (*p* < 0.05).

In our study of left atrial strain parameters, we observed that LASr, LAScd, LASr-c and LAScd-c were significantly lower in the T2DM group compared to the control group (*p* < 0.001). Conversely, LASct-c was significantly higher in the T2DM group than in the control group (*p* < 0.001).

**Figure 3:**
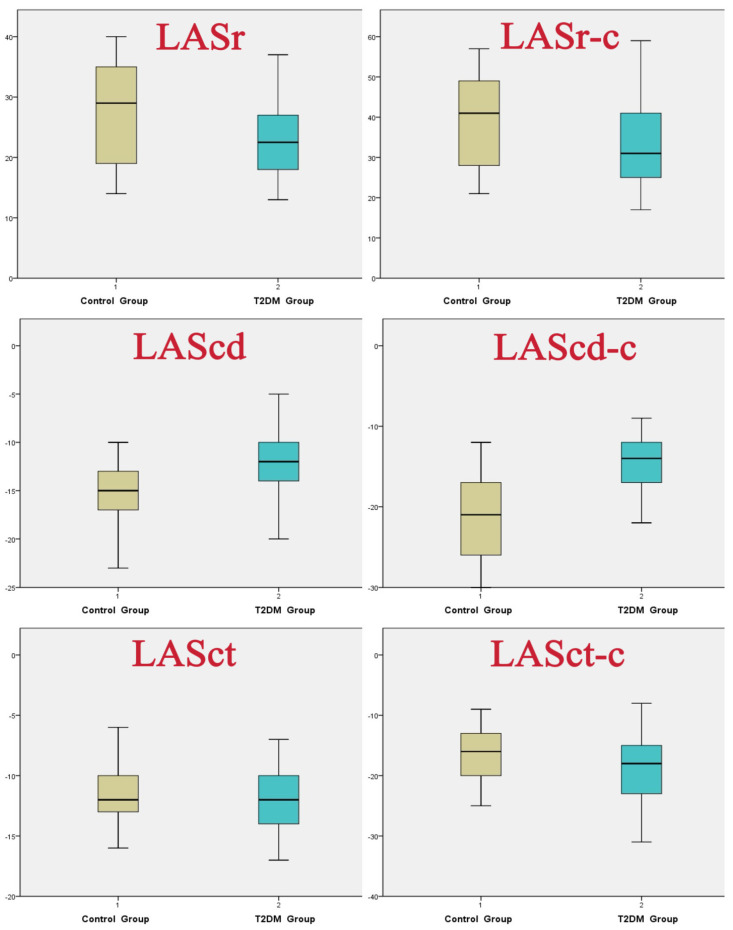
Comparison of 4D LAQ strain values between the two groups.

Notably, LASct did not show a significant difference between the two groups (Figure 3, Table 3).

### 3.4. Sensitivity Analysis

Among T2DM patients, 26 cases (41.9%) had normal weight (18.5–24.9 kg/m^2^), 30 cases (48.4%) were overweight (25–29.9 kg/m^2^), and 6 cases (9.7%) were underweight (none were obese). In controls, 34 cases (68%) were normal weight and 16 cases (32%) were overweight. To further explore the potential confounding effect of BMI, we performed a subgroup analysis restricted to normal-weight participants (BMI 18.5–24.9 kg/m^2^; 26 T2DM patients and 34 controls). The results remained consistent with the main analysis: T2DM patients exhibited significantly larger LA volumes, lower reservoir and conduit strains, and higher circumferential contractile strain compared to controls. These findings confirm that the observed LA alterations are not solely attributable to differences in BMI.

### 3.5. Univariate and Multivariate Linear Regression Analysis

Based on professional background knowledge and previous research findings, while excluding the influence of collinearity, age, BMI, glycated hemoglobin (HbA1c), E/A, e’/a’, and E/e’ were included in univariate linear regression analysis. According to the results of univariate linear regression, BMI, HbA1c, and E/e’ were gradually incorporated into a multivariate linear regression model. The structural analysis revealed that HbA1c and E/e’ were independently negatively correlated with LASr and LASr-c; HbA1c and E/e’ were independently positively correlated with LAScd; BMI, HbA1c, and E/e’ were independently positively correlated with LAScd; and HbA1c was independently negatively correlated with LAScd-c (*p* < 0.05) (Table 4).

### 3.6. Inter-Observer and Intra-Observer Variability

The assessment of inter-observer and intra-observer variability in the 4D echocardiography data revealed good intraclass correlation coefficients (ICCs). The ICCs for inter-observer variability of left atrial (LA) 4D longitudinal strain values (LASr, LAScd, LASct) and circumferential strain values (LASr-c, LAScd-c, LASct-c) ranged from 0.73 to 0.96. Concurrently, the ICCs for intra-observer variability ranged from 0.80 to 0.98.

## 4. Discussion

Previously, echocardiographic studies focusing on the diabetic mellitus (DM) population primarily examined left ventricular structure and function [17,18]. However, the atrioventricular coupling has been demonstrated to be a significant predictor of cardiovascular morbidity and mortality in DM patients. Each phase of the left atrial (LA) cycle plays a crucial role in atrioventricular coupling, thus highlighting the importance of accurately assessing LA phasic function over the past decade. LA function has three phases: reservoir during systole, conduit during early diastole [19], and booster pump during late diastole. LA reservoir function considers LA relaxation and compliance, moderated by LV systolic function. LA conduit function is closely associated with LV diastolic function, including the suction energy dependent on LV relaxation and LV chamber stiffness. LA contractile function represents intrinsic LA contractility and LV end-diastolic compliance and pressure [20]. The failure of any of the three LA phasic functions (reservoir, conduit or contractile) can potentially lead to LA dilatation, left ventricular diastolic dysfunction, atrial fibrillation, and ultimately, the development of heart failure with preserved (or even reduced) ejection fraction [21].

Several studies have accumulated evidence regarding LA volume and function changes in T2DM patients. Atas et al. [22] found that diabetic patients exhibited reduced reservoir and contractile function, while their conduit function remains similar to that of healthy controls. Tadic et al. [23] demonstrated an increase in LA volume, alongside a reduction in both reservoir and conduit function, although contractile function was enhanced in T2DM patients. However, the LA wall is composed of intricately intermingled longitudinal and circumferential bundles. The specific changes in LA longitudinal and circumferential strain across different cardiac phases remain uncertain. The recently developed 4D LAQ echocardiography may provide valuable quantitative and objective parameters to assess LA function. This technique offers better geometry assumption and higher temporal and spatial resolution, which may facilitate analysis of dynamic events such as the relaxation phase and isovolumic contraction [24]. Therefore, further exploration of T2DM patients using this novel 4D echocardiographic technique is warranted.

Left atrial size was initially assessed using M mode or conventional two-dimensional echocardiography by measuring the left atrial anteroposterior diameter (LAD) at end systole. LAD is easy to obtain and highly reproducible, making it suitable for routine clinical practice and large-scale studies. However, the left atrium is an asymmetrical three-dimensional structure, and a single linear dimension, such as LAD, often fails to accurately reflect true left atrial size. The present study corroborates this notion: although no significant difference was observed in LAD between the two groups, left atrial volumes measured by 4D LAQ were significantly increased in diabetic patients. This finding indicates that LA volume can increase even when the anteroposterior diameter remains normal, indicating early geometric remodeling. Left atrial enlargement reflects a compensatory response to chronically elevated left ventricular filling pressure and long-standing diastolic dysfunction. The left atrial wall is thin and composed of short myocardial fibers, rendering it susceptible to both pressure and volume overload. When impaired left ventricular relaxation impedes blood outflow, left atrial pressure rises, leading to increased phasic volumes.

Regarding left atrial function, we observed reduced reservoir and conduit functions in diabetic patients, accompanied by a compensatory increase in pump function. This was reflected by lower LASr, LAScd, LASr-c, and LAScd-c, along with higher LASct-c. Although the mechanisms linking atrial dysfunction to glucose regulation are complex and largely unknown, several explanations are possible. Persistent hyperglycemia promotes the accumulation of advanced glycation end products in the cardiac interstitium and deposition of denatured substances beneath the endothelium. This process results in disordered cardiac energy metabolism, myocyte hypertrophy, diffuse fibrosis, and microcirculatory damage, ultimately impairing both myocardial diastolic and systolic function [25,26]. Such alterations reduce atrial compliance and active relaxation, directly compromising both reservoir and conduit functions. Concurrently, diabetic patients show progressive declines in E/A and e’/a’ ratios and an increase in E/e’ ratio, indicating left ventricular diastolic dysfunction. This impairs LV filling, increases residual LA blood volume, and elevates preload. Through the Frank–Starling mechanism, the left atrium compensates by enhancing active contractility to maintain adequate ventricular filling. However, with disease progression, LA contractile function may eventually decompensate, as suggested by previous investigations [27].

A key finding of this study was that circumferential strain parameters may be more sensitive than longitudinal strain in detecting early diabetic atrial dysfunction. This was supported by the observation that compensatory augmentation of pump function was first evident in circumferential strain (LASct c). This finding aligns with LA myocardial architecture: the external layer consists of circumferential bundles, while the internal layer comprises longitudinal bundles that splay both longitudinally and circumferentially upon attaching to the mitral leaflet. Longitudinal strain assesses shortening along the long axis, while circumferential strain evaluates contraction along the short axis circumference [28]. We hypothesized that subendocardial longitudinal fibers may be more vulnerable to hyperglycemia-induced fibrosis and microvascular damage, leading to early impairment of longitudinal contractile reserve. In response, the epicardial circumferential fibers may undergo compensatory hyperkinesis to preserve global LA ejection fraction, a phenomenon observed in other conditions associated with atrial remodeling [29]. Thus, circumferential strain may more directly reflect the mechanical status of the dominant myocardial layer. Our findings suggest that circumferential strain may serve as a valuable complementary parameter for the early detection of left atrial dysfunction, a hypothesis that warrants further investigation.

Multivariable linear regression analysis identified key independent determinants of left atrial function. Glycated hemoglobin was independently associated with multiple strain parameters, indicating that poor long-term glycemic control is an independent metabolic factor impairing LA function, findings consistent with Tadic et al. [23]. The E/e’ ratio, a well-validated marker of LV diastolic function and filling pressure as endorsed by the American Society of Echocardiography and the European Association of Cardiovascular Imaging [30], was independent correlated with LA reservoir and conduit function, highlighting the central role of LV diastolic dysfunction in LA impairment. In addition, BMI was independently associated with conduit function, suggesting an independent contribution from obesity-related hemodynamic changes. As Chirinos et al. [31] demonstrated, LA conduit function declines with increasing BMI, likely due to increased preload and afterload from obesity-related hypervolemia.

### 4.1. Clinical Implications and Future Perspectives

Left ventricular global longitudinal strain (GLS) has been widely used for cardiac function assessment in patients with diabetes. The 2023 ESC Guidelines recommend GLS for screening subclinical myocardial dysfunction in diabetic patients [32]. In addition, GLS has demonstrated significant value in coronary artery disease risk stratification, with reduced resting GLS being closely associated with increased coronary artery disease burden [33]. Given the close atrioventricular coupling, left atrial strain—as a sensitive marker of left ventricular filling pressure and atrial myopathy—may complement GLS to provide a more comprehensive evaluation for the early detection and risk stratification of diabetic cardiomyopathy. In addition, future studies should include larger reproducibility samples and ideally multi-center assessments to establish more robust estimates of measurement variability.

### 4.2. Limitations

This study has several limitations. First, the cross-sectional design precludes any conclusions about the prognostic value of LA strain parameters. Whether the observed abnormalities predict incident atrial fibrillation, heart failure, or cardiovascular events in T2DM patients remains to be determined and requires prospective longitudinal studies. Second, the exclusion of hypertensive, obese, and cardiovascular disease patients strengthens internal validity, but limits generalizability to broader T2DM populations. Third, although the modest sample size (*n* = 62) was adequately powered for primary comparisons, it may not suffice for extensive subgroup analyses. Fourth, 4D Auto LAQ is a novel technique lacking vendor-independent validation and established normal reference values, and its dependence on high image quality may introduce selection bias. Finally, NT-proBNP levels were not measured. Multicenter studies with larger cohorts and biomarker correlations are needed.

## 5. Conclusions

In this cohort of newly diagnosed, treatment-naive patients with type 2 diabetes mellitus, 4D Auto LAQ echocardiography detected early left atrial volume enlargement and subclinical functional changes, specifically impaired reservoir and conduit function, accompanied by a compensatory increase in circumferential contractile strain. These alterations were independently associated with glycemic control (HbA1c) and left ventricular filling pressure (E/e’), suggesting a direct pathophysiological link between hyperglycemia and atrial myopathy in the absence of glucose-lowering medications.

Notably, the discordant response between circumferential and longitudinal contractile strain raises the hypothesis that circumferential assessment may offer greater sensitivity for detecting early compensatory mechanisms. However, this observation requires confirmation in larger studies and, ideally, histological correlation.

Given the cross-sectional design, modest sample size, and lack of established normative values for 4D LA strain, our findings should be considered hypothesis-generating rather than definitive. Whether these early strain abnormalities predict incident atrial fibrillation or heart failure remains to be determined. Prospective multicenter studies with longitudinal follow-up are warranted to validate the clinical utility of 4D Auto LAQ and its incremental value over conventional parameters in diabetic patients.

## Figures and Tables

**Table 1:** Comparison of clinical parameters and biochemical tests.

Variables	Controls (*n* = 50)	T2DM (*n* = 62)	*p* Value
Age (year)	49.50 ± 8.44	52.03 ± 9.92	0.154
Gender (male)	26 (52%)	35 (56%)	0.704
BMI (kg/m^2^)	23.96 ± 3.23	27.48 ± 3.83	<0.001
HR (bpm)	72.56 ± 8.21	74.95 ± 7.89	0.120
SBP (mmHg)	122.40 ± 9.41	125.63 ± 10.61	0.095
DBP (mmHg)	73.16 ± 6.94	75.15 ± 7.18	0.142
FPG (mmol/L)	4.77 ± 0.84	7.87 ± 1.13	<0.001
HbA1c (mmol/L)	5.00 ± 0.97	8.01 ± 1.12	<0.001
CRP (mg/L)	2.37 ± 0.98	4.43 ± 1.31	<0.001
TG (mmol/L)	1.62 ± 0.76	2.28 ± 0.91	<0.001
TC (mmol/L)	5.36 ± 0.97	6.10 ± 1.14	<0.001
HDL-C (mmol/L)	1.48 ± 0.48	1.28 ± 0.34	0.014
LDL-C (mmol/L)	3.50 ± 1.01	4.00 ± 1.20	0.023
CREA (mmol/L)	70.66 ± 15.00	74.42 ± 13.83	0.171

BMI: body mass Index; HR: heart rate; SBP: systolic blood pressure; DBP: diastolic blood pressure; FPG: fasting plasma glucose; HbA1c: glycated hemoglobin A1C; CRP: C reactive protein; TG: triglycerides; TC: total cholesterol; HDL-C: high-density lipoprotein cholesterol; LDL-C: low-density lipoprotein cholesterol; CREA: creatinine.

**Table 2:** Comparison of conventional echocardiographic parameters.

Variables	Controls (*n* = 50)	T2DM (*n* = 62)	*p* Value
LVEDD (mm)	49.38 ± 4.92	50.55 ± 5.49	0.244
LAD (mm)	30.50 ± 4.62	32.11 ± 4.85	0.077
LVST (mm)	9.89 ± 1.17	10.24 ± 1.10	0.104
PWT (mm)	9.57 ± 1.01	9.92 ± 1.02	0.076
SV (mL)	43.44 ± 6.08	42.53 ± 5.13	0.393
LVEF (%)	60.10 ± 4.62	58.61 ± 5.00	0.108
MVE (cm/s)	73.76 ± 8.73	71.71 ± 8.26	0.205
MVA (cm/s)	77.14 ± 8.95	79.94 ± 9.10	0.104
E/A	0.96 ± 0.11	0.89 ± 0.11	0.001
e’ (cm/s)	8.98 ± 0.67	7.16 ± 0.61	<0.001
a’ (cm/s)	7.81 ± 0.61	8.12 ± 0.68	0.014
e’/a’	1.16 ± 0.11	0.90 ± 0.10	<0.001
E/e’	7.99 ± 0.67	9.98 ± 0.91	<0.001

LVEDD: left ventricular end diastolic diameter; LVESD: left ventricular end systolic diameter; LAD: left atrial diameter; IVST: interventricular septum thickness; PWT: posterior wall thickness; EF: ejection fraction; MVE: peak E-wave velocity of the mitral valve; MVA: peak A-wave velocity of the mitral valve; e’: early diastolic peak velocity of the septal mitral annulus; a’: late diastolic peak velocity of the septal mitral annulus.

**Table 3:** Comparison of 4D LAQ parameters.

Variables	Controls (*n* = 50)	T2DM (*n* = 62)	*p* Value
LAVmin (ml)	18.66 ± 3.78	22.13 ± 4.47	<0.001
LAVmax (ml)	44.76 ± 7.62	49.81 ± 8.11	0.001
LAVpreA (ml)	32.32 ± 5.04	36.85 ± 6.26	<0.001
LAVmaxI (ml/m^2^)	26.40 ± 4.29	30.97 ± 5.14	<0.001
LAEF (%)	55.44 ± 6.60	52.05 ± 6.14	0.006
LASr (%)	26.90 ± 8.12	23.21 ± 6.41	<0.001
LAScd (%)	−15.38 ± 3.51	−11.03 ± 2.91	<0.001
LASct (%)	−11.92 ± 2.53	−12.18 ± 2.65	0.146
LASr-c (%)	38.46 ± 11.23	33.47 ± 9.98	0.014
LAScd-c (%)	−21.98 ± 5.99	−14.81 ± 3.09	<0.001
LASct-c (%)	−16.48 ± 4.54	−18.65 ± 5.59	0.029

LAVmin: left atrial minimal volume; LAVmax: left atrial maximum volume; LAVpreA: left atrial volume preatrial contraction; LAVmaxI: left atrial maximum volume index; LAEF: left atrial emptying fraction; LASr: left atrial peak longitudinal strain of reservoir function; LAScd: left atrial peak longitudinal strain of conduit function; LASct: left atrial peak longitudinal strain of contractile function; LASr-c: left atrial peak circumferential strain of reservoir function; LAScd-c: left atrial peak circumferential strain of conduit function; LASct-c: left atrial peak circumferential strain of contractile function.

**Table 4:** Univariate and multivariate linear regression analysis.

	LASr				LASr-c			
Parameters	Univariate		Multivariate		Univariate		Multivariate	
	β	*p*	β	*p*	β	*p*	β	*p*
Age (years)	−0.136	0.237	—	—	−0.172	0.391	—	—
BMI (kg/m^2^)	−0.185	0.331	—	—	−0.218	0.279	—	—
HbA1c (%)	−0.695	<0.001	−0.349	0.007	−0.603	<0.001	−0.318	0.016
E/A	0.196	0.032	—	—	0.165	0.029	—	—
e’/a’	0.153	0.027	—	—	0.188	0.022	—	—
E/e’	−0.674	0.002	−0.322	0.013	−0.615	<0.001	−0.281	0.019
	**LAScd**				**LAScd-c**			
**Parameters**	**univariate**		**multivariate**		**univariate**		**multivariate**	
	**β**	***p***	**β**	***p***	**β**	***p***	**β**	***p***
Age (years)	0.105	0.365	—	—	0.201	0.538	—	—
BMI (kg/m^2^)	0.176	0.032	—	—	0.272	0.014	0.159	0.042
HbA1c (%)	0.551	<0.001	0.405	<0.001	0.478	<0.001	0.414	0.003
E/A	−0.188	0.047	—	—	−0.201	0.105	—	—
e’/a’	−0.165	0.054	—	—	−0.179	0.136	—	—
E/e’	0.419	<0.001	0.317	<0.001	0.387	<0.001	0.305	<0.001
	**LASct**				**LASct-c**			
**Parameters**	**univariate**		**multivariate**		**univariate**		**multivariate**	
	**β**	***p***	**β**	***p***	**β**	***p***	**β**	***p***
Age (years)	−0.137	0.703	—	—	0.226	0.639	—	—
BMI (kg/m^2^)	0.038	0.561	—	—	0.126	0.438	—	—
HbA1c (%)	−0.196	0.016	—	—	−0.611	<0.001	−0.279	0.015
E/A	−0.203	0.127	—	—	−0.215	0.272	—	—
e’/a’	−0.231	0.092	—	—	−0.190	0.168	—	—
E/e’	−0.233	0.013	—	—	−0.627	<0.001	—	—

LASr/LASr-c: left atrial peak longitudinal/circumferential strain of reservoir function; LAScd/LAScd-c: left atrial peak longitudinal/circumferential strain of conduit function; LASct/LASct-c: left atrial peak longitudinal/circumferential strain of contractile function; BMI: body mass Index; HbA1c: glycated hemoglobin A1C; E/A: the ratio of peak E-wave/peak A-wave velocity of the mitral valve; e/a: the ratio of early/late diastolic peak velocity of the septal mitral annulus; E/e’: the ratio of peak E-wave velocity of the mitral valve/early diastolic peak velocity of the septal mitral annulus.

## Data Availability

Due to certain restrictions, the data is temporarily not publicly available. Please contact the author if needed.

## Abbreviations

The following abbreviations are used in this manuscript:

T2DM	Type 2 diabetes mellitus
4D	four dimensional
LAQ	Left atrial quantification
BMI	body mass index
HR	heart rate
SBP	systolic blood pressure
DBP	diastolic blood pressure
FPG	fasting plasma glucose
HbA1c	glycated hemoglobin A1C
CRP	C reactive protein
TG	triglycerides
TC	total cholesterol
HDL-C	high-density lipoprotein cholesterol
LDL-C	low-density lipoprotein cholesterol
CREA	creatinine
LVEDD	left ventricular end diastolic diameter
LVESD	left ventricular end systolic diameter
LAD	left atrial diameter
IVST	interventricular septum thickness
PWT	posterior wall thickness
LVEF	left ventricular ejection fraction
MVE	peak E-wave velocity of the mitral valve
MVA	peak A-wave velocity of the mitral valve
e’	early diastolic peak velocity of the septal mitral annulus
a‘	late diastolic peak velocity of the septal mitral annulus
LAVmin	left atrial minimal volume
LAVmax	left atrial maximum volume
LAVpreA	left atrial volume preatrial contraction
LAVmaxI	left atrial maximum volume index
LAEF	left atrial emptying fraction
LASr	left atrial peak longitudinal strain of reservoir function
LAScd	left atrial peak longitudinal strain of conduit function
LASct	left atrial peak longitudinal strain of contractile function
LASr-c	left atrial peak circumferential strain of reservoir function
LAScd-c	left atrial peak circumferential strain of conduit function
LASct-c	left atrial peak circumferential strain of contractile function
